# Mechanistic Studies of Idiosyncratic DILI: Clinical Implications

**DOI:** 10.3389/fphar.2019.00837

**Published:** 2019-07-26

**Authors:** Jack Uetrecht

**Affiliations:** Department of Pharmaceutical Sciences, University of Toronto, Toronto, ON, Canada

**Keywords:** drug-induced liver injury, immune mediated, reactive metabolites, mitochondrial injury, bile salt export pump, inflammasome

## Abstract

The idiosyncratic nature of idiosyncratic drug-induced liver injury (IDILI) makes mechanistic studies very difficult, and little is known with certainty. However, the fact that the IDILI caused by some drugs is associated with specific HLA genotypes provides strong evidence that it is mediated by the adaptive immune system. This is also consistent with the histology and the general characteristics of IDILI. However, there are other mechanistic hypotheses. Various *in vitro* and *in vivo* systems have been used to test hypotheses. Two other hypotheses are mitochondrial injury and inhibition of the bile salt export pump. It is possible that these mechanisms are responsible for some cases of IDILI or that these mechanisms are complementary and are involved in initiating an immune response. In general, it is believed that the initiation of an immune response requires activation of antigen-presenting cells by molecules such as danger-associated molecular pattern molecules (DAMPs). An attractive hypothesis for the mechanism by which DAMPs induce an immune response is through the activation of inflammasomes. The dominant immune response in the liver is immune tolerance, and it is only when immune tolerance fails that significant liver injury occurs. Consistent with this concept, an animal model was developed in which immune checkpoint inhibition unmasked the ability of drugs to cause liver injury. Although it appears that the liver damage is mediated by the adaptive immune system, an innate immune response is required for an adaptive immune response. The innate immune response is not dependent on specific HLA genes or T cell receptors and may occur in most patients and animals treated with a drug that can cause IDILI. Studies of the subclinical innate immune response to drugs may provide important mechanistic clues and provide a method to screen drugs for their potential to cause IDILI.

## Introduction

Idiosyncratic drug-induced liver injury (IDILI) is a major problem for clinicians, drug developers, and regulatory agencies. If we could accurately predict which drug candidates were likely to be associated with a significant risk of IDILI and other serious idiosyncratic drug reactions, it would have a profound effect on drug development. If we could predict which patients are at increased risk, it would make it possible to safely use drugs that are associated with a significant IDILI risk. If we could effectively treat IDILI and prevent liver failure, it would make it a less severe threat. It is unlikely that any of these goals will be achieved without a better mechanistic understanding of IDILI.

## Mechanistic Clues From Clinical Characteristics

In order to understand the mechanisms involved in IDILI, the first step is to develop hypotheses based on clues provided by clinical cases of IDILI and then find ways to test those hypotheses. IDILI is idiosyncratic in that most patients will not develop IDILI when treated with a drug known to cause IDILI in some patients (Uetrecht and Naisbitt, 2013). Genetic studies have been performed, and the major genetic factor that has come up as a significant risk factor is an association with specific HLA genotypes (Daly and Day, 2012). That provides very strong evidence that IDILI is immune mediated, at least for the drugs for which such an association exists. For most drugs, there are simply too few cases available to investigators to determine if there is a strong HLA association. Given that IDILI caused by other drugs has similar characteristics, it suggests that most IDILI is immune mediated. It is actually surprising that such strong HLA associations exist because if the immune response is directed against drug-modified proteins, most reactive drugs or reactive metabolites bind to many different proteins such that there would be multiple drug-modified peptides that have the potential to be presented by different HLA molecules. For example, the reactive metabolite of isoniazid binds to amino groups (Metushi et al., 2012), and most proteins contain at least one lysine. Isoniazid-induced IDILI has not been associated with a specific HLA genotype, and yet evidence suggests that it is immune mediated (Metushi et al., 2014a; Metushi et al., 2014b). Although the IDILI caused by several drugs is associated with a specific HLA genotype, if a patient with the required genotype is treated with the associated drug, it is unlikely that they will develop serious IDILI. For example, if a patient who is B*5701, which is a risk factor for flucloxacillin IDILI, is treated with flucloxacillin, there is less than a 1/500 chance that they will develop IDILI. Therefore, there must be other risk factors. Another significant risk factor appears to be the T cell receptor repertoire (Ko et al., 2011), but this is difficult to study. Another genetic factor that appears to increase the risk factor for IDILI independent of which drug is involved is a missense variant in PTPN22 which is also associated with an increase in the risk of various autoimmune diseases (Cirulli et al. 2019). This genotype appears to increase the risk of IDILI independent of the drug involved, and this also suggests that most IDILI is immune mediated.

An immune mechanism also explains the idiosyncratic nature of IDILI, e.g., we are familiar with the fact that other immune responses such as an allergy to peanuts are idiosyncratic. There is also ample evidence that other types of idiosyncratic adverse drug reactions that have similar characteristics as IDILI, e.g., skin rash, are immune mediated (Uetrecht and Naisbitt, 2013). Another characteristic of IDILI is a delay between starting the drug and the onset of clinical signs of liver injury. This delay is typically about 1–3 months (Chalasani et al., 2015), but it can be longer, especially for autoimmune IDILI, which often has a delay of more than a year (deLemos et al., 2014). The onset of IDILI can even be a month after discontinuation of the responsible drug (Keisu and Andersson, 2010). There are exceptions, especially with telithromycin (Clay et al., 2006) and the IDILI associated with the fluoroquinolones (Orman et al., 2011). This may indicate that the mechanism is different for these drugs. A delay i.An important characteristic of IDILI is that drugs associated with serious IDILI are always associated with a higher incidence of mild liver injury that often resolves despite continued treatment with the drug (Watkins, 2005). This is referred to as adaptation. However, if the injury is immune mediated, this resolution must involve immune tolerance. This is presumably why rechallenge in mild cases of IDILI does not always lead to rapid onset of liver injury; immune tolerance has already intervened.

IDILI is often said to be dose independent; nothing is dose independent! What is true is that, in general, the mechanism of IDILI is different from the mechanism of the therapeutic effect of the drug. An exception would be for immune modulators such as checkpoint inhibitors for which the mechanism of IDILI is related to their therapeutic effect. Therefore, there is no reason that the dose response curve should be in the same dose range, and it may be shifted to the left of the therapeutic dose response curve. However, the typical dose of a drug is about 10^20^ molecules, and it is axiomatic that a dose can always be found below which no one will develop IDILI. In fact, drugs given at a low dose are less likely to cause IDILI. An empirical cutoff is about 10 mg/day, i.e., if a drug is given at a total daily dose of 10 mg/day or less, it is unlikely to be associated with a significant incidence of IDILI (Uetrecht, 1999).

The histology of most hepatocellular IDILI is typical of an immune reaction with a monocytic inflammatory infiltrate similar to that of viral hepatitis (Foureau et al., 2015). The difference is that, on average, there are fewer NK cells in the inflammatory infiltrate of IDILI than in viral hepatitis.

IDILI is often also associated with a positive lymphocyte transformation test (Victorino and Maria, 1985). However, this is not always the case, and it appears to be drug-dependent (Whritenour et al., 2017). Another finding can be antidrug antibodies and/or autoantibodies (Kenna et al., 1984; Metushi et al., 2014a). Such studies are difficult to perform, so in most cases such studies have simply not been performed. Even IDILI caused by herbal products can be associated with anti-epoxide hydrolase antibodies, which suggests that it is immune mediated and caused by a reactive epoxide metabolite that modifies the epoxide hydrolase (Teschke et al., 2016).

These characteristics clearly suggest that most IDILI is immune mediated. Therefore, mechanistic studies should focus on testing the immune hypothesis with additional studies to elucidate the steps leading to the induction and resolution of immune responses to the drugs involved. There may be exceptions, and it is also possible that other toxic mechanisms contribute to the induction of an immune response. Therefore, it is reasonable to test other plausible hypotheses.

## IDILI Models

It is virtually impossible to prospectively study serious IDILI in humans, and retrospective studies make it difficult to separate cause from effect. Therefore, as with most areas of biomedical research, it is important to use models to study the mechanisms of IDILI.

### *In Vitro* Models

If IDILI is immune mediated, it is unlikely that it can be reproduced in a test tube; immune reactions involve many different types of cells, in multiple locations, and they evolve over time. For example, a plausible sequence of events leading to IDILI starts with reactive metabolite formation in hepatocytes, which results in drug-modified proteins and causes the release of danger-associated molecular pattern molecules (DAMPs) from hepatocytes. The DAMPs activate antigen-presenting cells that have taken up the drug-modified proteins. The antigen-presenting cells migrate to lymph nodes and spleen, which are designed to provide optimal conditions for interaction between the antigen-presenting cells and the few T cells that recognize specific drug-modified hepatic proteins. The activated T cells, both helper T cells, and cytotoxic T cells return to the liver under the influence of chemokines released by the hepatocytes and activated macrophages, and these T cells mediate the liver injury. These events also activate other cells such as T regulatory cells that usually prevent extensive damage to the liver. The mechanism is likely even more complex than this. Although this sequence of events cannot be duplicated *in vitro*, it may be possible to reproduce some aspects of the mechanism of IDILI *in vitro*. However, it would be inappropriate to infer the mechanism of IDILI from the results of *in vitro* studies unless the predictions from the *in vitro* studies are consistent with the characteristics of IDILI in patients.

#### *In Vitro* Cytotoxicity Models

The simplest models are *in vitro* models that try to relate cytotoxicity with IDILI risk. Many of the early studies used HepG2 cells because they are readily available and easy to culture (Atienzar et al., 2014). However, they have very limited capacity to metabolize drugs, and there is a large amount of evidence that most IDILI is caused by reactive metabolites of a drug rather than by the drug itself (Evans et al., 2004; Park et al., 2005). There are many other differences between HepG2 cells and normal hepatocytes. An alternative is to use fresh or frozen human hepatocytes. A relatively recent study claimed a true positive predictive rate of 50–60%, which is not very impressive, and the IDILI categorization of the majority of the drugs is quite questionable (Xu et al., 2008). Concentrations up to 100 times Cmax were used in the study; therefore, the mechanism in the assay is unlikely to be related to the mechanism of IDILI in humans. Even fresh human hepatocytes are not normal and quickly lose their ability to metabolize drugs. In the past, hepatocytes have been cultured in a two-dimensional culture, but it was discovered that hepatocyte spheroids retain function much longer than in a two-dimensional culture (Bell et al., 2016). Culture techniques have advanced, and there are several new platforms being used to study hepatocytes in combination with other cells (Underhill and Khetani, 2018). However, given that drugs that cause IDILI do not cause significant hepatocyte toxicity in most humans *in vivo*, it seems naïve to expect that hepatocyte cytotoxicity in culture would be able to predict the risk of IDILI, and they add little to our mechanistic understanding. There could be a correlation between overt cytotoxicity and the release of DAMPs, which are believed to be involved in the induction of an immune response; however, there is little evidence to support such a hypothesis.

#### Mitochondrial Injury

Although evidence strongly suggests that most IDILI is immune mediated, there could be exceptions, or some other mechanism may contribute to the induction of an immune response. Mitochondria control cell death and can be the source of DAMPs, which, in turn, can stimulate an immune response (Banoth and Cassel, 2018). Therefore, involvement of mitochondria is a plausible hypothesis. It has been proposed that mitochondrial injury is a common mechanism of IDILI (Dykens and Will, 2007). Although many assays have been used, respiration of isolated mitochondria (Hynes et al., 2006) and toxicity in the glucose/galactose model (Marroquin et al., 2007) are probably the most common. Many companies screen drug candidates for mitochondrial damage using these methods and claim that they provide value in predicting IDILI risk (Rana et al., 2018). However, strange test drugs were used in these studies. In particular, the study by Rana et al. classed the topical antibacterial agent, hexachlorophene, and rosiglitazone, which rarely, if ever, causes IDILI as positive DILI drugs. Worse, they class phenformin, which does inhibit complex I of the mitochondrial electron transport chain and was withdrawn because it causes sometimes-fatal lactic acidosis as a DILI drug, even though it also rarely, if ever, caused IDILI. Androgens such as danazol cause DILI with very different characteristics that can be predicted by their mechanism of action, and mitochondrial toxicity is unlikely to be involved. Procarbazine is a toxic anticancer alkylating agent; again, its toxicity is unlikely to involve uncoupling of oxidative phosphorylation as suggested. Few of the drugs classically associated with a relatively high risk of IDILI were tested, which is the risk that the pharmaceutical industry is presumably trying to predict. The concentrations used in these assays are typically much higher than the Cmax, and it is unlikely that the mechanism of toxicity at these high concentrations is the same as the mechanism of IDILI. Some drugs are concentrated in the liver, and it is possible for some drugs that higher concentrations would be appropriate, but no drug-specific information was used to justify higher concentrations. It is also possible that these drugs are also concentrated by hepatocytes in culture so that concentration adjustment would not be required; however, there is generally no data available to make it possible to use the appropriate concentrations.

As mentioned, even if inhibition of the mitochondrial electron transport chain does not directly lead to DILI, it could be involved in the induction of an immune response. It is interesting to note that there is a rationale for expecting that inhibition of the mitochondrial electron transport chain could actually decrease the risk of IDILI. Specifically, metformin has been reported to decrease the production of reactive oxygen species (ROS) by mitochondria, which is important for the activation of inflammasomes (Zhang et al., 2019). In contrast, the binding of rotenone to complex I is somewhat different from that of metformin, and it is reported to increase the production of ROS (Matsuzaki and Humphries, 2015). Rotenone has also been reported to cause activation of inflammasomes, which may be an important part of the mechanism of IDILI as discussed below (Martinez et al., 2017).

The mitochondria in animal cells are similar to those in human cells, and it should have been possible to reproduce DILI in animals if mitochondrial injury were the mechanism of DILI. The only published animal model of DILI that was developed to test the mitochondrial injury hypothesis involved a mitochondrial superoxide dismutase heterozygote, which was more sensitive to liver injury caused by drugs such as troglitazone (Ong et al., 2007); however, others have not been able to duplicate the findings in this model (Fujimoto et al., 2009). It was reported that the combination of rotenone and isoniazid was synergistic in killing hepatocytes *in vitro* (Lee et al., 2013). Rotenone is a classic inhibitor of complex I, and isoniazid inhibits complex II, either directly or through its hydrazine metabolite. It was proposed that other inhibitors of complex I would increase the risk of isoniazid-induced IDILI. When we tested this combination in mice, it was synergistic; all of the animals died (Cho et al., 2019). However, there was no evidence of liver injury. At slightly lower doses of rotenone, the animals survived, but again, there was no evidence of liver injury. When we tested the combination in our impaired immune tolerance model, which is described later, isoniazid did cause mild liver injury, but the injury was not significantly increased by the nonlethal doses of rotenone. This demonstrates the danger of using *in vitro* results to infer the mechanism of toxicity *in vivo*.

Without a clear link between the *in vitro* results and IDILI that occurs in humans, the *in vitro* results do little to test the hypothesis that mitochondrial injury is a common mechanism of IDILI. Furthermore, given that drugs that inhibit complex I of the mitochondrial electron transport chain do not cause IDILI, and IDILI rarely has characteristics that would be expected with inhibition of the mitochondrial electron transport chain such as lactic acidosis, it seems unlikely that such inhibition is a mechanism of IDILI. Yet, this is the basis for one of the most common assays of mitochondrial function, i.e., oxygen uptake by isolated mitochondria. It is possible that other mechanisms of mitochondrial injury are involved in the mechanism of IDILI. Acetaminophen-induced DILI does involve mitochondrial injury, but it is not idiosyncratic (Hinson et al., 2010). Inhibition of mitochondrial DNA synthesis (Montessori et al., 2003) and mitochondrial protein synthesis (McKee et al., 2006) do cause liver injury, but it is not limited to the liver, and it is not idiosyncratic. The one drug that causes IDILI for which there is strong evidence of mitochondrial injury is valproic acid (Stewart et al., 2010). Valproic acid is a branched aliphatic carboxylic acid. It is branched at the site of ß-oxidation of fatty acids; therefore, it inhibits fatty acid metabolism, leading to microvesicular steatosis. It also commonly leads to lactic acidosis, which is characteristic of mitochondrial injury. Furthermore, mutations in POLG, which codes for a mitochondrial DNA polymerase, are a risk factor for valproic acid-induced IDILI (Stewart et al., 2010). Another characteristic that is unique to valproic acid-induced IDILI is that infants are at much greater risk than adults (Bryant and Dreifuss, 1996). Thus, there are several lines of evidence that valproic acid-induced IDILI does involve mitochondria, but it has characteristics different from most IDILI, and the apparent mechanism would probably not be detected by the commonly used screening methods used to predict IDILI risk. In summary, the evidence does not support inhibition of the mitochondrial electron transport chain as a mechanism of IDILI. There may be other mechanisms of mitochondrial injury that cause or promote IDILI as mentioned later, but there is insufficient evidence to support such a hypothesis.

#### BSEP Inhibition

It is known that a complete genetic deficiency of the bile salt export pump (BSEP) leads to cholestatic liver injury and liver failure (Whitington et al., 1994). It is characterized by an increase in serum alkaline phosphatase but a normal γ-glutamyl transferase. It has been proposed that inhibition of BSEP by drugs is a common mechanism of IDILI, and drug candidates are commonly screened for their ability to inhibit BSEP (Morgan et al., 2010). The composition of bile acids is different in rodents than in humans with a higher percentage of the more polar and less toxic taurine conjugates in mice and more of the more toxic glycine conjugates in humans (Perwaiz et al., 2003). This complicates the development of a valid rodent model to study BSEP inhibition, and it makes it more difficult to test the hypothesis that BSEP inhibition leads to IDILI. Milder genetic deficiencies do not lead to serious liver injury (Lam et al., 2010). One reason for this is there are several mechanisms to compensate for a decrease in BSEP activity (Köck et al., 2014; Cheng et al., 2017). Given that the IC50 of most drugs that are reported to inhibit BSEP is greater than the Cmax of the drug, it seems unlikely that these drugs would increase bile acid concentrations sufficiently to cause liver injury. Even if there were significant inhibition at Cmax, bile acids could be cleared as the concentration of the drug decreased unless the drug had a long half life, and the drug concentration remained close to Cmax between dosing. The intracellular free concentration of drug may be higher than the serum-free concentration, but without data to correct for this, such a scenario cannot be assumed. If the drug also inhibited other compensatory mechanisms, it might be sufficient to lead to toxic concentrations of bile acids. Furthermore, in some cases such as troglitazone, it is a metabolite rather than the parent drug that has the greatest effect on BSEP (Funk et al., 2001). In my opinion, the correlation between BSEP IC50s and the risk of IDILI is not very good, and most of the drugs implicated do not cause cholestatic IDILI. Chan et al. found that simple physical properties of a drug candidate were better predictors of IDILI risk than BSEP inhibition (Chan and Benet, 2018). However, it is plausible that the mechanism of IDILI for a few drugs does involve inhibition of BSEP and other bile salt transporters, either directly or through the release of DAMPs that help to initiate an immune response. Given the caveats in the interpretation of *in vitro* BSEP inhibition data, it is important to determine if a drug causes an increase in serum bile acids in humans and, in turn, if the increase in serum bile acids is associated with liver injury. This has been done with bosentan. It was found that serum bile acids were elevated in patients with bosentan-induced liver injury, and co-treatment with glyburide, which also inhibits BSEP but is associated with a relatively low risk of IDILI, increased the incidence of bosentan-induced liver injury (Fattinger et al., 2001). It would have been even more compelling if serum bile acids were measured before the onset of liver injury to make sure that they were involved in the cause of the injury and not the result of liver injury.

#### Inflammasome Model

If most IDILI is immune mediated, it begs the question of how a drug or its metabolite activates the immune system. It appears that a common mechanism of immune activation involves activation of inflammasomes (Kubes and Mehal, 2012). These cytosolic protein complexes, present in immune cells, especially macrophages, activate caspase-1, which, in turn, converts pro-IL-1ß and pro-IL-18 to their active forms. Inflammasomes are activated by DAMPs. We found that with two pairs of drugs, telaprevir/boceprevir and dimethyl fumarate/ethacrynic acid, the drugs that caused a significant incidence of skin rash, i.e., telaprevir and dimethyl fumarate, activated inflammasomes, and those that are not associated with a significant incidence of rash, i.e., boceprevir and ethacrynic acid, did not (Weston and Uetrecht, 2014). These drugs are chemically reactive and presumably do not require bioactivation in order to cause an immune-mediated adverse reaction. In contrast, presumably one reason the liver is a common site of idiosyncratic drug reactions is because it is the site of most reactive metabolite formation. Hepatocytes can form reactive metabolites, and these reactive metabolites can cause hepatocyte damage leading to the release of DAMPs, but hepatocytes lack significant inflammasome activity. Therefore, we incubated drugs that cause IDILI with human hepatocyte spheroids and then determined whether the supernatant from this incubation could activate inflammasomes in THP-1 cells, a macrophage cell line (Kato and Uetrecht, 2017). We found that amodiaquine is easily oxidized to a reactive metabolite by THP-1 cells and directly activated inflammasomes in these cells. In contrast, nevirapine, another drug associated with a high incidence of IDILI, did not directly activate THP-1 cells, but the supernatant from incubation of nevirapine with hepatocytes did, and addition of aminobenzotriazole (a cytochromes P450 inhibitor) to the hepatocyte culture prevented the supernatant from activating inflammasomes in the THP-1 cells. We found that the supernatant from incubation of troglitazone and tolcapone, which are associated with IDILI, activated inflammasomes in THP-1 cells, but the supernatant from incubation of pioglitazone did not (Mak et al., 2018). The supernatant from an incubation of entacapone also activated THP-1 cells, even though it is not associated with the same risk of IDILI as tolcapone. In this case, the major differentiating feature between tolcapone and entacapone is that, unlike tolcapone, treatment of PD-1^−/−^ mice with anti-CTLA-4 and entacapone did not lead to an increase in T regulatory cells, while tolcapone treatment led to a brisk increase in T regulatory cells.

### Animal Models

Animal models are very important for mechanistic studies in most areas of biomedical research. However, as with humans, idiosyncratic drug reactions are also idiosyncratic in animals; therefore, treatment of animals with drugs that can cause IDILI in humans does not generally cause liver injury. Most animal models of IDILI have involved treatment of animals with very large doses of drug, and in some cases, acute liver injury is observed. However, this is very different from the characteristics of IDILI in humans and presumably involves different mechanisms (Ng et al., 2012). One exception is amodiaquine. Treatment of mice with amodiaquine leads to a delayed-onset liver injury that is mediated by NK cells (Metushi et al., 2015a). To be useful, the animal model must involve a mechanism at least similar to that which occurs in humans.

#### Inflammagen Model

If most IDILI is immune mediated, it seems likely that factors that stimulate the immune system would increase the risk of IDILI. A model was developed based on this hypothesis (Roth et al., 2003). Specifically, it is claimed that treatment of animals with lipopolysaccharide (LPS), which binds to toll-like receptor (TLR)-4, unmasked the ability of drugs to cause IDILI. However, the characteristics of this model are very different from those of IDILI: it is acute rather than delayed, and it is mediated by neutrophils rather than mononuclear cells. We have not been able to reproduce IDILI in animals by stimulation of the immune system through TLRs or other methods. We even immunized mice with amodiaquine-modified hepatic proteins prior to oral treatment with amodiaquine, but we found that this immunization actually prevented the mild injury that occurred with amodiaquine alone (Mak and Uetrecht, 2015a). This immunization led to an increase in T regulatory cells and myeloid-derived suppressor cells, which presumably explains the protective effects of immunization. The results in animals are consistent with the observation that patients with inflammatory conditions such as ulcerative colitis and whose livers are exposed to large amounts of LPS are not at significantly increased risk of IDILI when treated with drugs that can cause IDILI. It appears that the liver usually has the ability to prevent a damaging inflammatory response.

#### Impaired Immune Tolerance Model

The dominant immune response in the liver is immune tolerance. This is essential because the liver is exposed to multiple bacterial antigens and inflammatory molecules from the intestine. Therefore, instead of trying to stimulate the immune system, an alternate strategy is to impair immune tolerance. A major development in the treatment of cancer has been the development of antibodies that block immune checkpoints and promote an immune response to tumors (Pardoll, 2012). The two major immune checkpoints are cytotoxic T-lymphocyte-associated protein-4 (CTLA-4) and programmed cell death protein-1 (PD-1). Using an analogous strategy, we used a combination of PD-1^−/−^ mice and treatment with anti-CTLA-4 antibodies to impair immune tolerance. The first drug that we studied in this model was amodiaquine because it causes mild immune-mediated liver injury even in wild-type mice. We found that treatment of PD-1^−/−^ mice with a combination of anti-CTLA-4 and amodiaquine led to a delayed-onset liver injury with characteristics similar to IDILI in humans (Metushi et al., 2015b). Specifically, the histology was characterized by a mononuclear inflammatory infiltrate and piecemeal necrosis. Unlike in wild-type mice, the injury did not resolve with continued treatment, and it led to decreased liver function with an increase in serum bilirubin. The injury was mediated by CD8 T cells because depletion of these cells prevented the injury (Mak and Uetrecht, 2015b). We tested this model to determine if it might be a general method to unmask the ability of drugs to cause IDILI. It was successful in all of the drugs that are associated with IDILI that we tested, and it differentiated troglitazone from pioglitazone and tolcapone from entacapone (Mak and Uetrecht, 2015c; Mak et al., 2018). However, in all cases, the injury was significantly milder than with amodiaquine, and in contrast to amodiaquine, the injury resolved despite continued treatment with the drug. Immune checkpoint inhibitors also increase the risk of IDILI caused by co-administered drugs when they are used clinically for the treatment of cancer (Suzman et al., 2018). However, it is unlikely that impaired immune tolerance plays a significant role in IDILI. If it did, such patients would be at increased risk of autoimmune disease and to IDILI caused by multiple drugs, and this does not appear to be the case. This model may be useful to predict which drug candidates are likely to be associated with an increased risk of causing IDILI; however, it is unlikely to be perfect because of species difference in drug metabolism. Probably, more important is that this model will allow us to study the early steps in the induction of an immune response that in the rare individual can lead to liver failure as mentioned in section 3.1.4. A better mechanistic understanding of IDILI could also lead to better treatment options.

#### Innate Immune Responses to Drugs

The observation that the IDILI caused by several drugs is associated with specific HLA genotypes suggests that it is mediated by the adaptive immune system (Daly and Day, 2009). This is consistent with the liver histology of IDILI in humans (Foureau et al., 2015) and the observation that depletion of CD8 T cells was protective in the amodiaquine/impaired immune tolerance model. However, an innate immune response is required in order to activate the adaptive immune system. Innate immune responses are not HLA and T cell receptor restricted and may occur in most patients and animals. For example, clozapine is associated with idiosyncratic agranulocytosis, which occurs with an incidence of about 2/1,000 patients treated. However, most patients treated with clozapine have an immune response with a transient increase in serum C-reactive protein and IL-6 and a paradoxical neutrophilia (Pollmacher et al., 1996). We have also found evidence of an innate immune response to nevirapine in wild-type mice treated with nevirapine (unpublished observation). If drugs that cause IDILI generally cause a subclinical innate immune response, it would provide clues to the initial steps in the immune response that only rarely leads to liver failure. Such immune responses might even provide a way to screen drug candidates, in both animals and humans, for their potential to cause a more serious adaptive immune response.

## Human Studies

The impaired immune tolerance model described above will allow us to perform detailed mechanistic studies of IDILI. However, it is just a model. It is essential that the findings in the model be compared with immune responses that occur in humans. For example, Warrington et al. performed lymphocyte transformation tests on patients with isoniazid-induced liver injury (Warrington et al., 1982). In those patients with small increases in ALT, the test was only positive to isoniazid-modified proteins (human serum albumin modified by adduction with isonicotinic acid), but in patients with severe isoniazid-induced liver injury, their lymphocytes also responded to parent drug. This provides clues to what chemical species initiated the immune response and how the immune response evolved in more severe cases. We found that patients treated with isoniazid who had a small increase in ALT also had an increase in Th17 cells and cells expressing IL-10, presumably Tr1 cells (Metushi et al., 2016). The studies of patients being started on clozapine was mentioned above, most of whom developed a mild immune response to the drug. If it is true that drugs that cause IDILI commonly cause subclinical immune responses in patients that evolve over time and usually resolve with immune tolerance, studies of these immune responses and how they correlate with IDILI risk would go a long way to provide a better mechanistic understanding of IDILI.

## Summary

The various major mechanistic hypotheses are summarized in **Table 1**. There is a large amount of evidence to support the hypothesis that most IDILI is immune mediated; however, there may very well be exceptions. It is dangerous to extrapolate from the results of *in vitro* studies to infer the mechanism of IDILI without confirming that the *in vitro* results are consistent with clinical observations. For example, evidence suggests that mitochondrial injury, at least inhibition of the mitochondrial electron transport chain, is not involved in the mechanism of IDILI. In contrast, BSEP inhibition may be involved in the mechanism of some cases of IDILI, but there is insufficient clinical data to provide confidence that this is a common mechanism, or to determine exactly what role BSEP inhibition plays in the overall mechanism. Activation of inflammasomes is an attractive mechanism for the initiation of an immune response that can lead to IDILI, but it is too early to know if this is a general mechanism. Inhibition of immune tolerance has led to an animal model of IDILI with characteristics very similar to clinical IDILI, but again, it is important to make links between the model and what happens in humans. The fact that there is an association between the IDILI caused by several drugs and specific HLA genotypes suggests that the injury is caused by an adaptive immune response, and that is consistent with the impaired immune tolerance model and liver histology. However, an adaptive immune response likely requires an initial innate immune response, which would not be restricted by HLA genotypes and T cell receptor repertoires. A study of the innate immune response in both animals and humans to drugs that cause IDILI may provide important information about the initial steps in the initiation of IDILI, and it could also provide a method to predict IDILI risk. Multiple factors may be involved in the mechanism IDILI, and the factors may be different for different drugs. Significant progress has been made in the last decade in our understanding of the mechanisms of IDILI, but the immune response is very complex, other mechanisms are likely to contribute in some cases, and there is still a long way to go.

**Table 1:** Summary of the major hypotheses for the mechanism of idiosyncratic drug-induced liver injury (IDILI).

	Plausibility/supporting evidence	Evidence against/lack of clinical evidence
Adaptive immune mechanism as primary cause	• HLA associations • General characteristics such as delay in onset and rapid onset on rechallenge and sometimes onset after drug discontinuation • Histology • Anti-drug and/or autoantibodies • + Lymphocyte transformation tests • Simple explanation for idiosyncratic nature • Impaired immune tolerance unmasks the ability of drugs to cause liver injury in mice, and the injury has many of the characteristics of clinical IDILI. • Immune checkpoint inhibitors increase the risk of IDILI with co-administered drugs in humans • Increased risk of IDILI in patients with PTPN22 genotype that is associated with several autoimmune diseases	• Features such as HLA association and anti-drug antibodies have only been observed in a limited number of cases; however, for several reasons, this is not surprising. • Features such as anti-drug or autoantibodies could be the result of toxicity rather than the cause. • Liver failure does not occur in the impaired immune tolerance animal model. • Some IDILI such as that caused by telithromycin is not associated with a delay in onset. • There very well may be exceptions, i.e., even if most IDILI is immune mediated, some IDILI with similar characteristics may involve a different mechanism.
Innate immune response as a contributory mechanism	• Cell injury/stress and innate immune response is required for induction of adaptive immune response. • Reactive metabolites can lead to activation of the inflammasome.	• There has been very little characterization of the innate immune response to drugs that cause IDILI.
Reactive metabolites as primary cause	• There is a large amount of circumstantial evidence to suggest that reactive metabolites are responsible for most IDILI.	• IDILI characteristics suggest that direct cytotoxicity is not the primary mechanism of most IDILI. • IDILI is not readily reproducible in animals.
Reactive metabolites as a contributing mechanism	• See above • Reactive metabolites can both act as haptens and also cause cell damage leading to the release of DAMPs. • Some IDILI is associated with anti-drug antibodies, which suggests that the immune response was induced by reactive metabolite-modified proteins. • It is clear that some chemically reactive drugs can cause an immune response, e.g., penicillin, and reactive chemicals can induce an immune response, e.g., contact hypersensitivity. • The fact that the liver is the primary site of reactive metabolite formation may be the reason that it is a common target for idiosyncratic drug reactions.	• It is very difficult to directly prove that a reactive metabolite is responsible for IDILI caused by a specific drug, and association does not prove causation. • Not all reactive metabolites appear to be associated with the same risk of IDILI. • Some drugs that cause IDILI such as ximelagatran do not appear to form a reactive metabolite.
Inflammatory mechanism as a primary cause	• Plausible mechanism • Animal model	• With few exceptions, patients with inflammatory conditions are not at increased risk of IDILI. • Characteristics of the animal model are very different from clinical IDILI.
Inflammation as a contributory mechanism	• Activation of antigen-presenting cells is required to induce an adaptive immune response, and inflammation should activate antigen-presenting cells.	• See above • The immune system is a constant balance between activation and tolerance, and inflammation also upregulates tolerogenic responses. • The response to agents such as LPS is rapidly down-regulated with repeated exposure.
Primary BSEP inhibition mechanism	• Bile salts are toxic. • Severe hereditary deficiency in BSEP leads to liver failure.	• Characteristics of most IDILI are different from those of liver failure due to BSEP deficiency.
BSEP inhibition as a contributory mechanism	• Attractive hypothesis for a mechanism to release DAMPs required for immune activation. • Many drugs that cause IDILI inhibit BSEP.	• There are many compensatory mechanisms that decrease the effects of BSEP inhibition; therefore, inhibition of multiple pathways may be required to cause liver injury. • Correlation between risk and BSEP inhibition is not very convincing, and the inhibition IC50 is usually > Cmax. • Little support from clinical data, e.g., for drugs in which BSEP inhibition is important the drug would be expected to cause an increase in serum bile salts.
Primary mitochondrial mechanism	• Mitochondria are vital to cell function. • Many drugs that cause IDILI inhibit mitochondrial electron transport or cause some other type of mitochondrial damage *in vitro*. • Valproate IDILI is associated with evidence of mitochondrial dysfunction.	• With the exception of valproate IDILI, clinical IDILI is not associated with characteristics of mitochondrial dysfunction such as lactic acidosis or steatosis. • Drugs that do cause significant inhibition of the mitochondrial electron transport chain, e.g., metformin do not cause IDILI. • Virtually all of the supporting evidence is *in vitro*, most at concentrations > Cmax.
Mitochondrial injury as a contributory mechanism	• Inhibition of the mitochondrial electron transport chain could cause cell stress leading to immune activation. • Mitochondria contain molecules that when released act as DAMPs that would cause immune activation.	• Drugs that inhibit the electron transport chain such as metformin do not increase the IDILI risk of co-administered drugs. • With the exception of valproate IDILI, no clinical characteristics suggest mitochondria involvement.

See the text for details and references. (BSEP, bile salt export protein; DAMPs, danger-associated molecular pattern molecules; LPS, lipopolysaccharide.)

## Author Contributions

JU is the sole author of this work.

## Funding

The author’s research is funded by a grant from the Canadian Institutes of Health Research.

## Conflict of Interest Statement

The author declares that the research was conducted in the absence of any commercial or financial relationships that could be construed as a potential conflict of interest. The reviewer GK declared a past co-authorship with the author to the handling editor.
